# Impact of early childhood caries and its treatment under general anesthesia on orofacial function and quality of life: A prospective comparative study

**DOI:** 10.4317/medoral.21611

**Published:** 2017-04-08

**Authors:** Valérie Collado, Hélène Pichot, Caroline Delfosse, Caroline Eschevins, Emmanuel Nicolas, Martine Hennequin

**Affiliations:** 1 Université Clermont Auvergne, CROC, F-63000 Clermont-Ferrand, France; 2 CHU Clermont-Ferrand, Service de soins spécifiques, F-63003 Clermont-Ferrand, France; 3 Sanitary and Social Agency of New Caledonia, Nouméa, New Caledonia; 4 Department of Pediatric Dentistry, Dental School, Lille 2 University, Place de Verdun, 59000 Lille, France

## Abstract

**Background:**

Early Childhood Caries (ECC) has become a major public health concern worldwide, mostly affecting children from disadvantaged families in increasingly severe forms. This condition has been frequently reported to alter children’s nutrition, growth and general development. It negatively impacts their quality of life, through painful episodes and severe eating difficulties. While this period is crucial for oral praxes development, the impact of dental state on oro-facial functions is poorly documented. This study evaluated the impact of ECC and its treatment under general anesthesia on oro-facial functions and quality of life in pre-school children.

**Material and Methods:**

The dysfunction and quality of life scores from 25 children with ECC were evaluated before treatment (T0), one month (T1) and three months after treatment (T2), using the Nordic Orofacial Test-Screening (NOT-S) and the Early Childhood Oral Health Impact Scale (ECOHIS), respectively, in comparison with 16 caries-free children. The number and extent of inter-arch dental contacts were also observed.

**Results:**

The pre-operative higher NOT-S score observed in children with ECC decreased to reach the control level at T2. The mastication item was the most affected in the ECC group throughout the study. Their mean ECOHIS score also significantly decreased post-operatively and differences remaining between both groups were no longer clinically relevant. In addition, in children with ECC, values of functional inter-arch surfaces tended to increase over the follow-up period.

**Conclusions:**

Oro-facial functions and quality of life, altered by ECC, could be restored through a conservative treatment approach. Relations between dental state, orofacial functions and particularly chewing, and nutrition should be investigated further.

** Key words:**Early childhood caries, children, orofacial dysfunction, general anesthesia, quality of life, occlusal contacts.

## Introduction

Dental caries have been considered as the most common chronic health problem in childhood, and dental care is the most prevalent unmet health need in children (1,2). In particular, Early Childhood Caries (ECC), which mostly affects children from disadvantaged families in increasingly severe forms, has become a major public health concern worldwide (3,4). Early onset of ECC is not easy to identify for parents and primary health care providers (5), and children mostly visit a healthcare professional in the case of pain and/or infections when the disease is already well established. Treatment under general anesthesia (GA) is thus often the only viable option to ensure quality dental care in pre-cooperative children (6). Its positive impact on child health and well-being is now recognized (7).

The psychosocial, physical and functional consequences of ECC have often been evoked through acute infectious episodes, sleep loss, irritability due to pain, and also eating difficulty (8,9). In particular, the disease has an impact on children’s nutrition, growth and development. Affected children are known to display a higher risk of developing iron deficiency anemia than caries-free children (10). Body Mass Index (BMI) is also affected, although the population with ECC does not have a specific weight distribution, and the link between ECC and obesity is controversial (11). Paradoxically, to date, the relation between dental state and chewing efficiency and their consequences on nutrition and growth have not been investigated in children. However, even in ancient societies, the role of mastication in nutrition was recognized, and recent studies have indeed implied a scientific link between the oral stage and certain subsequent metabolic mechanisms (12).

In adults and adolescents, interactions between dental status and chewing efficiency were also investigated in different situations and in various groups of patients (13,14). In particular, adults with multiple untreated carious lesions experienced chewing deficiency, manifested by a decrease in food comminution related to the number of functional posterior teeth, food selection and functional deteriorations in oral-health related quality of life (15). Other studies have correlated the extent of inter-arch contact areas with masticatory performance (16,17), suggesting that eating difficulties may not only be due to pain in the presence of carious lesions. However, improving the inter-arch relationship by replacing natural teeth with prosthetic dentures did not fully restore masticatory function (18). Chewing function is regulated by neuromotor control through dental sensory receptors (19), but it is still unknown whether conservative dental treatment, which would preserve most of this sensory information, could lead to more efficient chewing function. Beyond this question, the real role of teeth on nutrition is of interest.

In children, the role of teeth in orofacial function development and maturation, and especially chewing, has been given very little attention, in particular in the preschool period. However, early childhood is a key period for acquiring praxes involved in all orofacial functions. Young children with extensive caries may therefore constitute good models for studying the role of dental state on the development of orofacial functions. A comprehensive screening instrument, the Nordic Orofacial Test-screening (NOT-S) assessing different domains within the orofacial functions, was developed (20). This evaluation tool has been used in several studies among different groups of patients, including very young children (21). However, very few included the dental state of their subjects. So far, only one study including children and adults with ectodermal dysplasia has demonstrated a negative impact of oligodontia on orofacial function (20,22). Another study on schoolchildren and adolescents with orthodontic needs indicated that orofacial dysfunctions are related to the deterioration of masticatory performance (23). Within the same age range, masticatory performance was also positively correlated with dental status and with posterior inter-arch contacts (24,25). On the contrary, data obtained from younger children failed to link masticatory performance to the inter-arch contact area (26). Indeed, factors affecting masticatory performance may differ according to the developmental stage of the individual. In the case of early child-hood caries, it is possible that praxes involving tongue and lip muscles, still present in young children, could partially offset the deterioration of their dental state. The long-term effect of such possible compensation on orofacial function maturation has yet to be investigated.

This study aimed at evaluating the impact of early childhood caries and its conservative rehabilitation under general anesthesia on the frequency and the nature of orofacial dysfunction using the NOT-S questionnaire, and children’s Oral Health-Related Quality of life (OHRQoL). The evolution of inter-arch functional contacts participating in mastication was also observed.

## Material and Methods

This observational comparative prospective study, designed in accordance with Good Clinical Practice (International Clinical Harmonisation, 1996), was approved by the local ethical committee (CECIC, IRB Number 5044). Parents, or legal guardians, and children received oral and written information explaining the study design and the possible benefits and constraints related to their participation. Their informed consent was obtained.

- Participants

The study group consisted of preschool children (2-6 years), healthy or with mild systemic disease (ASA1 or ASA2, respectively), in need of dental care under GA due to their young age and the number of procedures needed. Inclusion took place during a pre-operative visit (T0) at the Special Dental Care Unit of the University Hospital of Clermont-Ferrand (France) between September 2009 and July 2011. Children with disability, geographical barriers preventing follow-up visits and/or without social security insurance were excluded. The required sample size was estimated from a preliminary study that measured the NOTS score in an ECC group before and after GA. The mean values of the NOTS-score decreased from 1.5 ± 1.2 (before GA) to 0.25 ± 0.71 (one month after GA). Calculations with the epiR package 0.9-30 were based on a difference of 1.25 and a common standard deviation value of 0.9. It indicated the need for at least 22 participants (α = 5%, β = 10%).

The control group consisted of children from 2 to 6 years with healthy teeth, included between September and December 2009. These children were recruited within the research laboratory circle, as it was difficult to include children without caries in the hospital setting. The provenance of participants is not supposed to influence the impact of dental health on orofacial function and quality of life results. Statistical analyses (student-t test, sample size calculation for unequal sample size design) were performed to calculate the adequate sample size for the control group. Consequently, 16 children were included.

Medico-social and oral descriptive data are given in  Table 1 for both groups of children. Both groups were similar in age (t-test) and gender (Fisher exact test). Five out of the 41 children included were slightly over the age limit (71 months) to meet the ECC definition according to the American Association of Pediatric Dentistry (AAPD) (3). Two children belonged to the control group (both were 72 months) and three to the experimental group (72, 74 and 75 months respectively). Since these three children had also multiple carious cavities in deciduous teeth, they were included in the “ECC group” in order to simplify the analysis. Furthermore, one child in the control group and four children with ECC had at least one permanent erupting tooth (first molars or central incisors) at T0, and one of them had two cavities in permanent teeth.

**Table 1:**
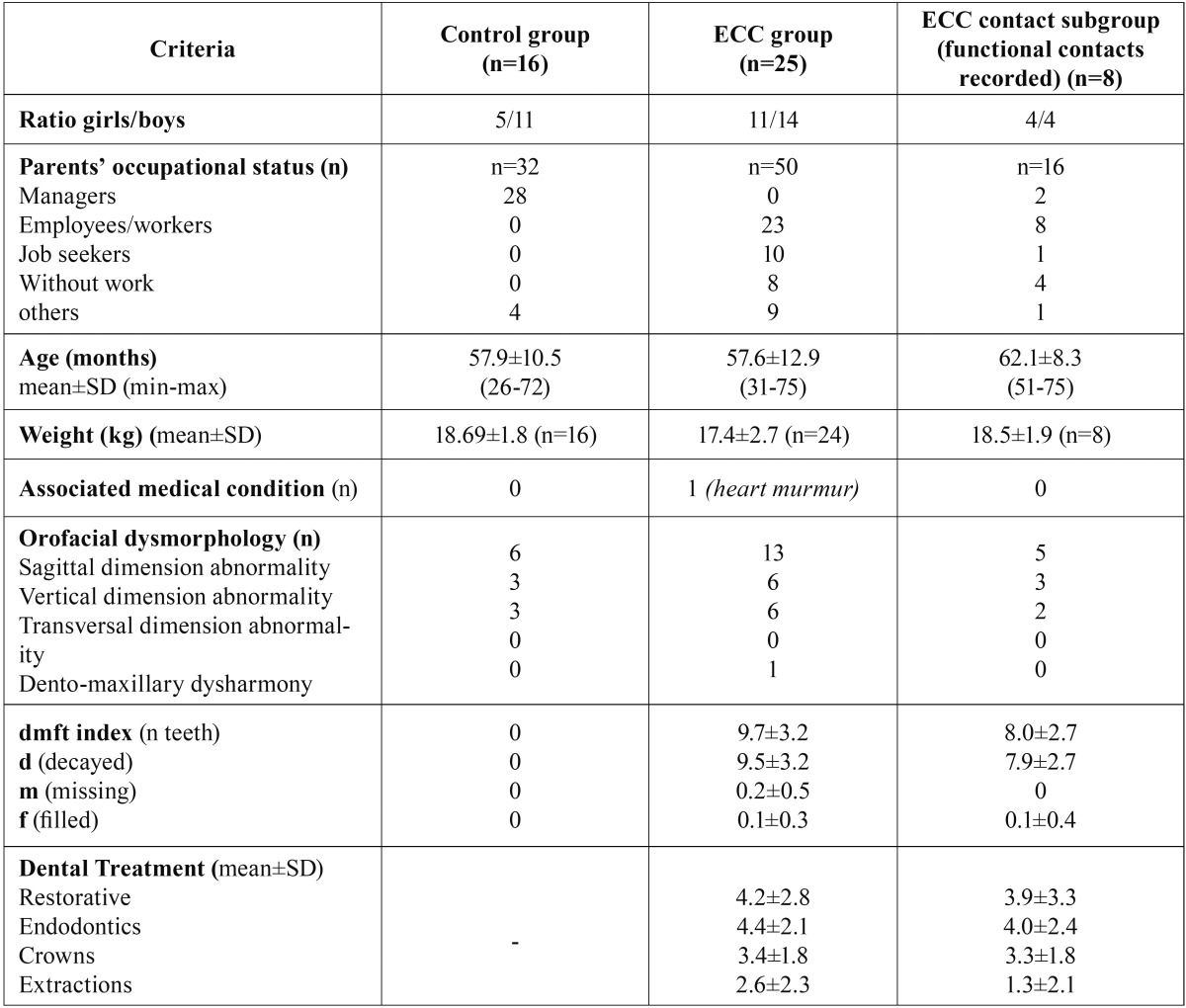
Medico-social and oral descriptive data for each group of children.

To describe the children’s dental state, the mean decayed/missing/filled teeth index (dmft) was calculated for both groups. The overall mean dmft score was 9.72±3.22 (min: 4; max: 16) for children with ECC, which corresponds to severe-ECC according to the AAPD3. In children with ECC, an average of 11.9±3.7 teeth per child were treated and 2.6±2.3 teeth were extracted under GA. Treatment under GA was as conservative as possible, in order to maintain sensory information from teeth.

Moreover, a causal relationship has been recognized between dento-maxillary dysmorphology and orofacial dysfunction (27). Thus, to evaluate the real impact of extractions and conservative treatments provided under GA on orofacial function, the presence or absence of orofacial dysmorphology was checked using a simplified version of the Index of Orthodontic Treatment Need (IOTN). This tool specifically measured sagittal, vertical, and transversal dimension abnormalities, as well as dento-maxillary disharmony and crowding (28). No difference was found between either group of children regarding the presence of dysmorphology (Fisher-exact test).

- Data collection

For the control group, data collection was carried out during a single evaluation, as the study criteria were not expected to change significantly over the study period. For the ECC group, three visits included in the regular follow-up agenda were scheduled for each child: T0 (pre-operatively), T1 (one month after GA), and T2 (3 months after GA). Two practitioners of the Special Dental Care Unit carried out the consultations for both groups, each involving a clinical examination and an interview with the child and its parents. The data collected concerned socio-demographic information, the children’s general health status, oral health information and the study evaluation criteria described further on. Both investigators met before and frequently during the course of the study to harmonize the recording method.

* Evaluation criteria

- Orofacial dysfunction score (NOT-S score)

The main evaluation criterion was the frequency of orofacial dysfunctions evaluated by the Nordic Orofacial Test-Screening (NOT-S), which consisted of a structured interview and a clinical examination (20). The French version of the NOT-S was used in this study (http://mun-h-center.se/en/Mun-H-Center/Mun-H-Center-E/NOT-S/). The interview contains 6 domains: (I) sensory function, (II) breathing, (III) habits, (IV) chewing and swallowing, (V) drooling, and (VI) dryness of the mouth. The clinical ex-amination includes 6 domains: [1] the face at rest, [2] nose breathing, [3] facial expression, [4] masticatory muscle and jaw, [5] oral motor function, and [6] speech. Each domain gives a maximum score of one point, even if several items within the same domain are scored positively. The total NOT-S score may vary from 0 to 12.

- Impact of dental caries on quality of life

The impact of ECC on child functioning, well-being and Oral Health-Related Quality of Life (OHRQoL) was investigated using a validated French version of the Early Childhood Oral Health Impact Scale (ECOHIS), especially adapted for preschool children (25).

ECOHIS relies on parental ratings of 13 items grouped into two main parts: part one is the child impact section and consists of four domains: child symptoms (1 item), child functions (4 items), child psychology (2 items), and child self-image and social interaction (2 items). Part two is the family impact section and consists of two domains: parental distress (2 items) and family function (2 items). The scale is scored using a simple Likert frequency type scale, with responses ranging from “Never” to “Very often” (equivalent to scores of 0 to 4) plus a “Don’t know” option (also scored 0). Item scores were simply added to create a total scale score that ranged from 0 to 52, with higher scores indicating greater impacts and/or more problems.

- Inter-arch functional contacts

The total number of “Functional Units” (TotFU) was defined as the number of pairs of antagonist teeth with at least one contact area during chewing. The child was asked to chew 2-3 cycles on a 200-μm articulating paper (29). The number of teeth from the lower arch that made at least one colored mark gave the number of TotFU. It was decided not to record potential new contacts from erupting permanent teeth. The maximum TotFU value recorded in fully dentate caries-free young children without dysmorphology was 20.

Functional occlusal Surfaces were also registered to specify the extent of the inter-arch contacts, by scanning an elastomeric impression of the inter-dental space with jaws in maximal intercuspidal occlusion. Placed in sitting position on the dental chair, with their head supported by the headrest, the children were trained to close their teeth in the correct position. The silicone paste was first placed with an impression gun on the mandibular arch and the child was encouraged to bite downwards in order to register dental occlusion. Each impression was prepared for scanning using the Silverfast® image analysis software. The OSC (Occlusal Scanning Control) software was then used to determine the surface area (in mm2) of the Total inter-arch Functional Surface (TotFS) for which the silicon thickness ranged from 0 (perforating surface) to 500 μm (near contact surfaces) (29). The impres-sion of potential permanent teeth was excluded from the analysis.

The number of Functional Units and Functional Surfaces was also analyzed separately for both Anterior (incisors and canines) and Posterior (molars) areas (respectively number of Anterior and Posterior Functional Units: AntFU and PostFU; Anterior and Posterior Functional Surfaces: AntFS and PostFS).

- Statistical Analysis

Data were collected and analyzed using IBM®SPSS® 19 software.

The analysis was performed for the 25 children with ECC, except for the inter-arch functional contact criteria for which only 8 complete datasets were obtained ( Table 1). This group of 8 children was called “ECC contact” subgroup and it was checked to ascertain whether it was not different from the whole ECC group regarding age, dmft index scores, ECOHIS and NOT-S scores (Student t-test, NS).

For each evaluation criterion, the comparison of average values of NOT-S and ECOHIS scores, the number of Functional Units (TotFU, AntFU and PostFU) and the Functional Surfaces (TotFS, AntFS and PostFS) was made between the ECC group and the control group at each evaluation time using a t-test. A paired t-test was used to compare each evaluation criterion within the same ECC group at the different evaluation times.

## Results

- Orofacial dysfunction score (NOT-S)

In the control group, no dysfunction was scored during the evaluation. The total mean level of dysfunction was significantly higher in the ECC group than in the control group at T0 (t test, *p*≤0.001) and at T1 (*p*≤0.05), but decreased significantly between T0 and T1 (*p*<0.01) so that no difference was observed any longer between the groups at T2 ( Table 2). Throughout the study, 18 children from the ECC group out of the 25 decreased their total NOT-S score, 6 maintained their score and one score was increased.

**Table 2:**
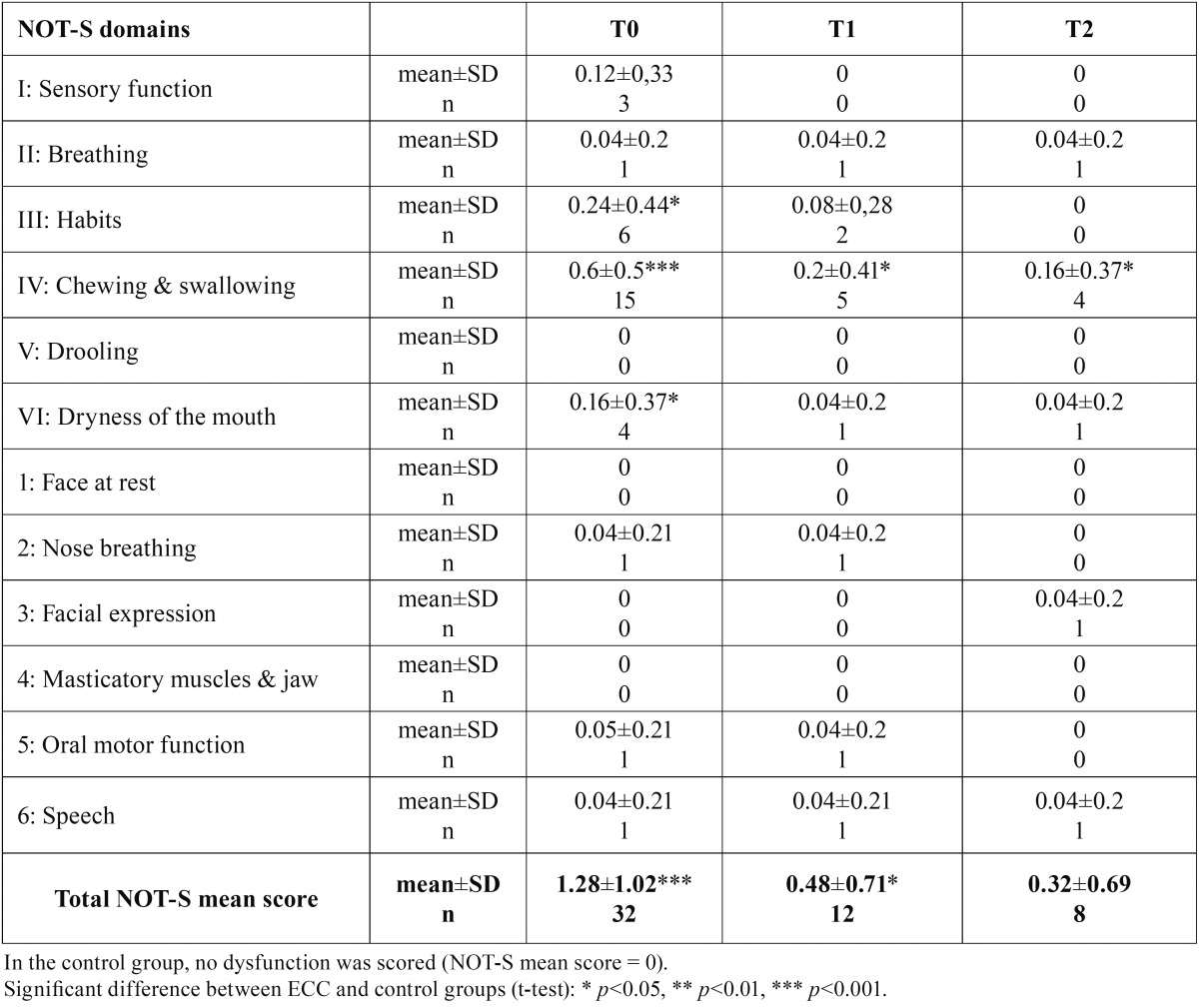
Comparison of Nordic Orofacial Test-Screening (NOT-S) mean scores between the control group and the ECC group throughout the study.

**Figure 1:**
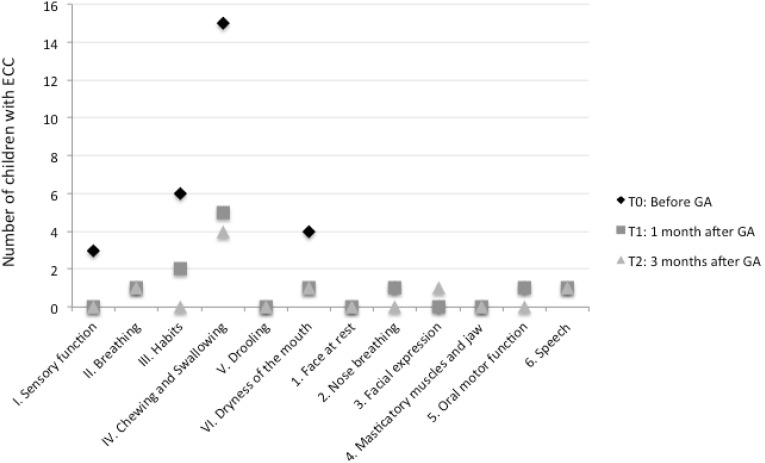
Frequency of dysfunctions for each domain of the Nordic Orofacial Test-Screening throughout the study.

The item-by-item intergroup comparison of NOT-S mean scores is shown in  Table 2. Item IV (chewing and swallowing) was the most prevalent throughout the study (15/25 children at T0, 5/25 at T1 and 4/25 at T2) (Fig. 1). NOT-S items V (drooling), 1 (face at rest) and 4 (masticatory muscle and jaw) were never scored as altered domains.

- Impact on quality of life (ECOHIS) 

In the control group, no quality of life item was impaired. At T0, the mean ECOHIS score was significantly higher in the ECC group (11.04±7.67) than in the control group (*p*<0.001) ( Table 3). This score decreased significantly between T0 and T1 in the ECC group (*p*<0.001). However, the difference between groups remained significant until T2 (*p*<0.001 at T1 and *p*<0.01 at T2).

**Table 3:**
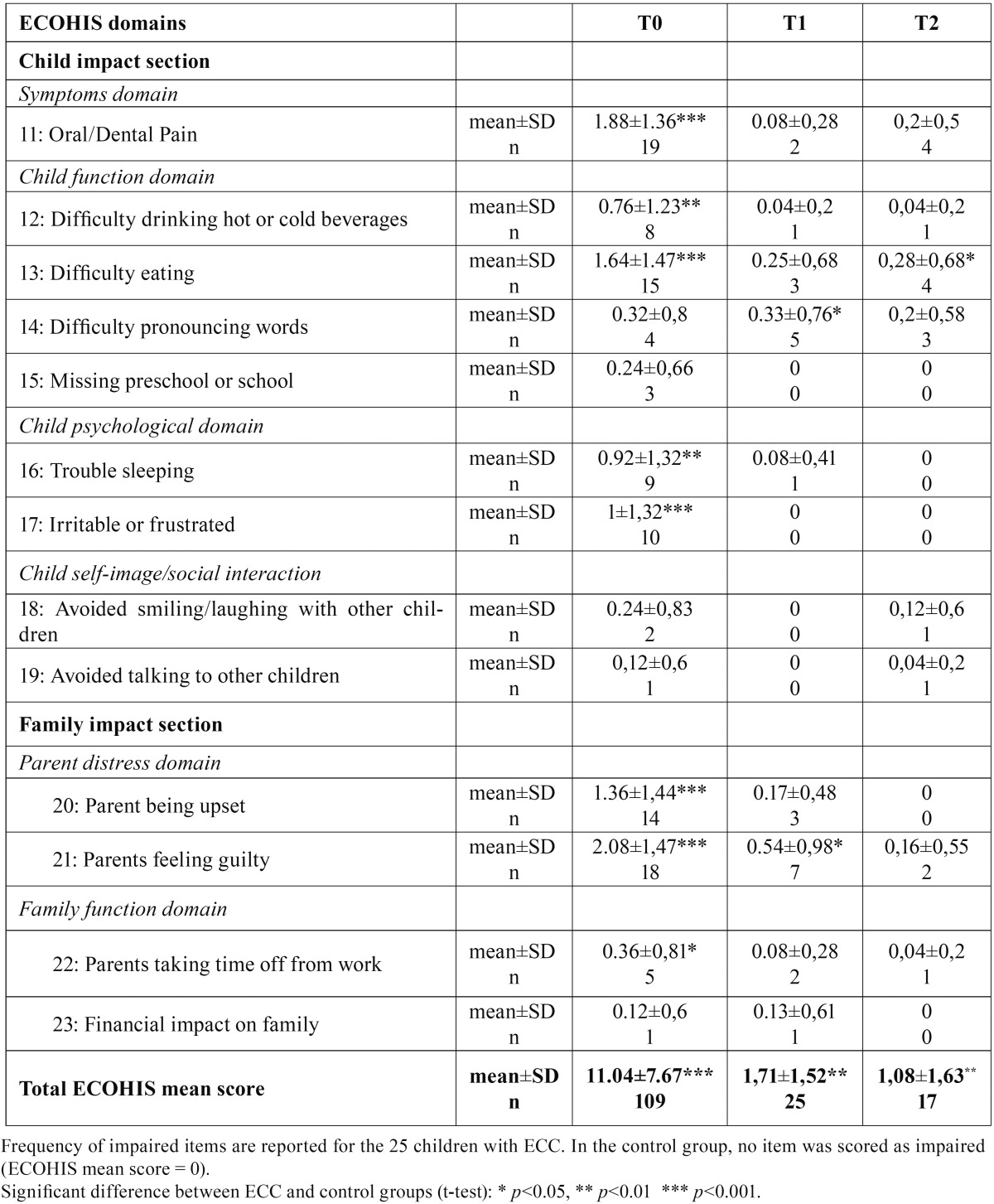
Comparison of Early Childhood Oral Health Impact Scale (ECOHIS) mean scores between the control group and the ECC group throughout the study.

In the ECC group, the score of 23 children out of 25 decreased between T0 and T1. At T1 and T2 respectively 7 and 14 children from the ECC group had an ECOHIS score of 0.

The item-by-item comparison is presented in  Table 3. The most frequent ECOHIS items cited by parents at T0 were “pain” (reported for 19/25 children), “eating difficulty” (15/25), “parents feeling guilty” (18/25) and “parents being upset” (14/25).

- Inter-arch functional contacts (FU and FS)

The mean number of TotFU was lower in the ECC group than in the control group at each evaluation time (*p*<0.05 at T0 and T2 and *p*<0.001 at T1). Post-operatively (T1 and T2), the mean number of AntFU remained significantly lower in the ECC group than in the control group (*p*<0.01 at T1 and *p*<0.05 at T2). However, the mean number of PostFU remained similar between groups and over time.

At T0, the mean values of inter-arch Functional Surfaces (TotFS, AntFS and PostFS) were not different between groups ( Table 4). The mean TotFS value was only significantly lower in the ECC group than that in the control group at T1 (*p*<0.05). In the ECC group, the mean TotFS, PostFS and AntFS values tended to increase after treatment, but the difference with preoperative values was significant only for the anterior area and only at T2 (*p*<0.05).

**Table 4:**
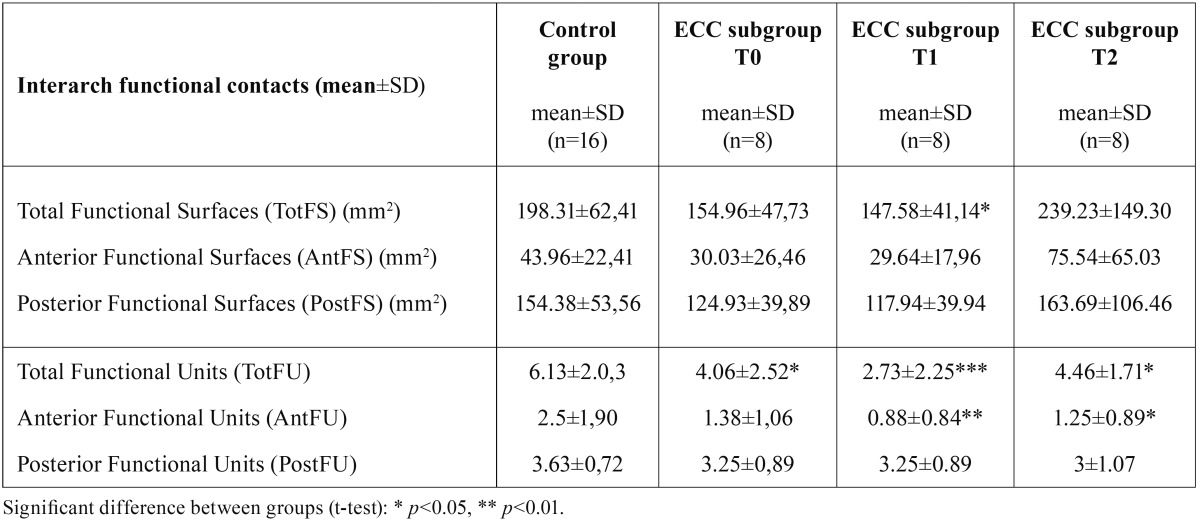
Comparison of Mean Interarch Functional Surfaces and Number of Functional Units throughout the study for both groups.

## Discussion

This study was the first step in exploring the links between dental condition and orofacial function in young children with extensive carious lesions. This analysis objectively assessed the negative impact of ECC on oral function, in particular on children’s ability to chew and swallow. It also confirmed the negative influence of ECC on children’s quality of life, consistent with recent literature data (7,9). Indeed, in our study, children with ECC experienced significant OHRQoL alterations in the symptomatic, functional and psychological domains and in the parents’ distress domain. After rehabilitation, the quality of life of children with ECC improved drastically compared to the preoperative evaluation. Although the differences between the two groups remained statistically significant after three months, they were no longer clinically relevant as children had become pain free and reported no eating difficulties ( Table 3). It is noteworthy that the ECOHIS scores remained lower than those obtained by other authors for the same population (7). The greater improvement of quality of life observed in our study may be due to some extent to the very conservative approach of treatments under general anesthesia, possibly restoring functional occlusal contacts, and thus sensory information from teeth.

In our study however, caution is required when interpreting the results concerning the impact of inter-arch contacts on function. Only a very small sample (n=8) could be analyzed because of the difficulty of obtaining an elastomeric impression in a correct occlusion position from these young children with potentially painful teeth. Nonetheless, children with ECC tended to have fewer pairs of antagonist teeth in contact and smaller contact surfaces before treatment than caries-free children, mainly due to tooth decay. During the few months after rehabilitation, occlusal contact surfaces tended to increase to reach the control group level, whereas less functional units remained due to extractions, particularly in the anterior area. One explanation could be that oral function is improved by the restoration of the anatomical support, in turn leading to progressive natural occlusal equilibration. However, additional proof of the benefits for orofacial function obtained from a more or less conservative therapeutic approach in this population is still needed (30). Indeed, our study sample was quite homogeneous regarding the extraction rate, and the limited number of participants in this study did not allow investigating the impact of treatment options on oral functions.

This study is the first to show that dental rehabilitation successfully restores orofacial function in young children, as early as three months after treatment. This result confirmed the capacity of the NOT-S questionnaire to evaluate the outcomes of oral rehabilitation (21). One domain related to “chewing and swallowing” was still highlighted post-operatively in ECC children, although the remaining scores were very low. A longer adaptation period may be necessary to restore or develop efficient masticatory function. Another issue is the intervention of a speech therapist to retrain the function and to assist the parents and children to change their feeding habits. Indeed, orofacial and chewing development may also be compromised in children with ECC because they are not exposed in the first years of childhood to a sufficiently broad range of food textures (31). When mastication is altered, parents probably adapt food choices to their child’s capacity and acceptance during food diversification. It is possible that these acquired feeding habits are not naturally modified after rehabilitation. Individual dietary behavior and habits could therefore be influenced in the long-term by early functional deficiencies. Moreover, in children with oral disease, producing a ready-to-swallow food bolus may be difficult and the state of the bolus when finally swallowed remains unknown. Additional studies are necessary to characterize the food bolus of children during their development, as well as the factors influencing bolus granulometry. The complete nutritional risk assessment of these children has not been documented either. Nevertheless, developing efficient mastication during childhood may lead to nutritional benefits in adulthood, as endocrinal pathways induced by the cephalic phase during mastication regulate satiation and bolus comminution, and lead to efficient nutrient uptake (32). Finally, the links between oral condition, oral function and nutritional state must be investigated further by taking a systematic clinical research approach. Assessment of young children’s masticatory efficiency, in relation with their dental state, is currently underway through food bolus particle size analysis.

This work is the first study to provide an objective assessment of the effect of early childhood caries on orofacial functions, as well as its negative impact on children’s quality of life. It also provides evidence of functional restoration through conservative dental treatment approach.

This population could be a good model to study the role of dental state in oral function development in children and underlying nutritional aspects.
